# In reply to: Interpreting antidepressant prescribing trends in Croatia

**DOI:** 10.3325/cmj.2026.67.325

**Published:** 2026-08

**Authors:** Katarina Gvozdanović, Roberto Mužić, Helena Orehovački, Danijela Štimac Grbić

**Affiliations:** 1 Department of Pharmacoepidemiology, Teaching Institute of Public Health, Zagreb, Croatia; 2 Croatian Institute of Public Health, Zagreb, Croatia; 3 Health Care Centre Zagreb – Centar, Zagreb, Croatia

We thank Zeeshan et al (1) for their comments on our article and for recognizing the importance of studying antidepressant prescribing patterns in Croatia. We also appreciate the opportunity to clarify several points regarding the interpretation of our findings. With regard to the short-term peak in prescriptions noted in March 2020, we proposed stockpiling as a possible explanation rather than establishing it as the cause, clearly stating that the peak in prescriptions “may reflect the stockpiling behavior at the beginning of the pandemic” and explicitly adding that this finding “should be interpreted with caution.” This interpretation is consistent with EU-wide collaborative work including Croatian data, in which an immediate increase in antidepressant dispensing at the beginning of the pandemic, followed by a decrease in the subsequent two months, was observed across almost all participating countries and regions and was considered potentially indicative of medication hoarding (2). We therefore maintain that proposing stockpiling as one plausible explanation, rather than an established cause, was a reasonable and appropriately hedged interpretation.

Similarly, based on our data, we cannot establish why fluvoxamine prescribing increased during the pandemic. Our statement that increased fluvoxamine consumption “could be linked to fluvoxamine’s potential ability to reduce the risk of hospitalization for patients with COVID-19” was intended to reflect the clinical perception and expectations current at the time not to address fluvoxamine’s actual effectiveness. In that period, there was considerable clinical interest in fluvoxamine as a potential COVID-19 treatment, based on emerging evidence regarding its possible clinical effects. Subsequent Croatian population-based evidence did not demonstrate a reduced risk of COVID-19-related hospitalization or mortality among outpatients prescribed fluvoxamine (3). We therefore accept that this later evidence does not support a protective effect of fluvoxamine in this population. However, whether a drug ultimately proved effective and whether expectations about its potential effectiveness shaped prescribing behavior at the time are distinct questions; our original statement addressed the latter. Nevertheless, we agree that this distinction could have been made more explicit.

Our original study was descriptive by design, intended to characterize national antidepressant prescribing patterns from 2017 to 2022, and was not designed as a causal evaluation of the pandemic’s effect. Consistent with this design, we stated that the observed trends could not simply be attributed to the pandemic, but that its potential influence could not be ignored. We appreciate the comment concerning interrupted time-series (ITS) analysis. Collaborative work using ITS methodology and including Croatian data has since provided a more formal assessment of pandemic-related changes in antidepressant dispensing across 11 European regions, supporting the view that the pandemic may have influenced antidepressant utilization while also demonstrating that its effects differed substantially by age, sex, and region (2).

Regarding benzodiazepine utilization, we agree that our data do not allow us to establish individual-level substitution between benzodiazepines and antidepressants, as we had no patient-level information on co-prescribing, switching, or treatment discontinuation. We accept that, as framed, our original explanation risked an ecological fallacy. High benzodiazepine utilization in Croatia has nonetheless been consistently documented in both national and comparative studies (4,5), and we offered our observation as contextual background that may be relevant to interpreting comparatively lower antidepressant use, rather than as evidence of a substitution effect. We did not intend to imply a causal or patient-level relationship.

We agree that future research should combine longitudinal ITS approaches with patient-level linkage of antidepressant and benzodiazepine prescribing data. Such analyses would allow investigation of switching, co-prescribing, treatment initiation, and discontinuation, and would provide a stronger basis for evaluating whether benzodiazepine use contributes to differences in antidepressant treatment patterns.

We believe these clarifications help place our findings within the appropriate context and further emphasize the need for more detailed pharmacoepidemiological studies of psychotropic medication use in Croatia.
